# Effect of bodyweight interval training on frailty status in older adults: a randomized controlled trial

**DOI:** 10.1007/s41999-026-01579-8

**Published:** 2026-08-12

**Authors:** Lucas Marques Vieira, Danilo Sales Bocalini, Clarcson Plácido Conceição dos Santos, Leandro Brasil Rego, Rodrigo Villar, Aylton José Figueira Júnior, Rodrigo Chaves da Silva, Ana Paula de Souza Lima, Ruy Barbosa Martins Calheiros Netto, Felício Savioli Neto, Taina Costa Nonato, João Paulo Pinto, Francisco Luciano Pontes Júnior

**Affiliations:** 1 School of Arts, Sciences and Humanities, University of São Paulo - Exercise Physiology and Aging Laboratory (EACH-USP), São Paulo, SP Brazil; 2 Federal University of Espírito Santo, Vitória, Espírito Santo Brazil; 3 Bahiana School of Medicine and Public Health, Salvador, Bahia Brazil; 4 University of Manitoba, Winnipeg, MB Canada; 5 São Judas Tadeu University, São Paulo, São Paulo Brazil; 6 Dante Pazzanese Institute of Cardiology, São Paulo, São Paulo Brazil

**Keywords:** Frailty, Older adults, Bodyweight exercise, Interval training, Primary health care, Randomized controlled trial

## Abstract

**Purpose:**

To evaluate the effect of bodyweight interval training on frailty status in older adults.

**Methods:**

A randomized controlled trial was conducted with 60 frail older adults (74.0 ± 6.0 years; 72.4% women). Participants were allocated to bodyweight interval training (BWIT = 30) for 24 weeks or to a control group (CG = 30) instructed to maintain their usual physical activities. Frailty status (Fried phenotype), physical-functional performance, cardiohemodynamic responses, and body composition were assessed at baseline and after 12 and 24 weeks. Longitudinal effects were analyzed using linear mixed-effects models and categorical between-group comparisons.

**Results:**

BWIT was associated with a higher probability of transition to less frail states over 24 weeks (p < 0.001), whereas the control group remained stable. Significant effects were observed in the intervention group (OR = 15.44; p = 0.006) and over time at 12 weeks (OR = 4.96; p = 0.005) and 24 weeks (OR = 15.24; p < 0.001). BWIT improved knee extension strength, five-times sit-to-stand performance, gait speed, and reduced blood pressure parameters (p < 0.001), with no relevant changes in body composition.

**Conclusion:**

BWIT was effective in modifying frailty status in older adults over 24 weeks and may represent a feasible and accessible strategy for frailty management in primary health care.

Trial registration: RBR-23t5jbh (Brazilian Registry of Clinical Trials), registered September 4, 2024, retrospectively registered.

**Supplementary Information:**

The online version contains supplementary material available at https://doi.org/10.1007/s41999-026-01579-8.

## Key summary points

**Aim:**

This study aims to evaluate the effect of bodyweight interval training on frailty status in older adults in primary health care.

**Findings:**

Bodyweight interval training increased the probability of transition to less frail states among older adults over 24 weeks, with improvements in physical-functional performance and reductions in blood pressure parameters. No significant changes were observed in body composition.

**Message:**

Bodyweight interval training may represent a strategy for frailty management in older adults in primary health care.

**Supplementary Information:**

The online version contains supplementary material available at https://doi.org/10.1007/s41999-026-01579-8.

## Introduction

Global population aging is not always accompanied by adequate health and functional status  [1]. This scenario may contribute to complex clinical states, such as geriatric syndromes [2], among which frailty is of particular relevance.

Declines across multiple physiological systems and reduced capacity to respond to stressors characterize frailty [3], a condition that presents high epidemiological magnitude, with a prevalence of 9.1% among community-dwelling older adults in Brazil and 17.4% globally, while pre-frailty is even more prevalent (51.8% of older adults in Brazil and 46% worldwide) [4–6]. This panorama is configured as one of the main contemporary challenges for health systems [7], requiring effective and feasible interventions in the context of primary health care [8, 9].

The presence of frailty in older adults is associated with increased risk of falls, dependency, hospitalizations, institutionalization, and mortality [7, 10, 11]. Recent evidence recognizes this syndrome as a dynamic condition, subject to changes over time [10], with frequent transitions between its states, with occurrence of both progression and reversal [12, 13], which reinforces its clinical relevance as a potentially modifiable condition in aging.

In this context, physical exercise stands out in the prevention and management of frailty [14, 15]. Approaches such as multicomponent exercise or strength exercises are widely investigated, contributing to the reduction of severity [16], functional gains [17, 18], higher strength levels [19], and reduction in fall risk [17, 19].

Although these types of programs present favorable effects, their implementation frequently involves structural and operational demands that require environments with greater availability of resources, specialized supervision, and the use of specific equipment [20], restricting applicability in primary health care [7, 21].

Given these limitations, bodyweight exercise emerges as a low-cost, potentially effective, and accessible intervention in combating frailty, capable of promoting clinical adaptations [22] with lower dependence on external resources and greater flexibility of application. This modality is configured as a physical training method that uses the individual’s own body weight as mechanical resistance, allowing systematic manipulation of its variables and favoring neuromuscular and functional adaptations in older adults [23, 24].

Despite the scarcity of studies that directly evaluate its impact on frailty status, recent research has identified significant effects in older adults with clinical conditions [24, 25], as well as benefits in physiological resilience in healthy older adults [26] and in functional capacity [27]. However, it remains undefined whether bodyweight training, specifically in an interval format, is effective in modifying frailty status in older adults.

Thus, the objective of this study was to evaluate the effect of bodyweight interval training on frailty status in older adults, and on physical-functional performance, cardiohemodynamic, and body composition outcomes.

## Methods

### Study conduct and ethical aspects

This is a randomized, controlled, parallel-group clinical trial conducted following the Consolidated Standards of Reporting Trials (CONSORT) guidelines [28]. The protocol was registered and published in the Brazilian Registry of Clinical Trials (ReBEC) under the identifier RBR-23t5jbh. This study was approved by the Research Ethics Committee of the School of Arts, Sciences and Humanities of the University of São Paulo (Approval No. 6764949), fully complying with the standards established by Resolution 466/2012 of the National Health Council (CNS). All stages of the research were conducted in accordance with the guidelines and principles of the Declaration of Helsinki [29].

### Participants

The sample related to the eligible population for this research consisted of older adults aged 60 years or older, with fully voluntary participation, of both sexes, recruited from primary health care, more specifically from the Basic Health Unit (UBS) Barcelona, located in Sorocaba (city in the State of São Paulo—Brazil). Volunteer recruitment was carried out at the Basic Health Unit, where older adults were invited to participate in the research through clinical referral after routine medical consultation, and through dissemination by the principal investigator in the reception area of the unit using an informational poster. Screening started in June 2024. Only older adults classified as frail were eligible for the study according to the criteria established by Fried et al. [3]. Individuals were not eligible for participation if they presented dementia, psychiatric disorders, mental disability, stroke with severe impairment, physical impairment that made mobility impossible, or did not meet the frailty classification.

### Randomization and allocation

The Study Randomizer software [30] was used to allocate each participant sequentially to one of the groups: BWIT group or control group (CG). Immediately after consent to participate and frailty screening, the principal investigator performed the randomization and allocation procedure using the software, individually with each participant. A homogeneous distribution between groups (1:1) was determined using age stratification (< 75 years and ≥ 75 years) and the permuted block algorithm with variable block sizes (block 4, block 6), thus avoiding predictability in allocation. In addition, allocation concealment was ensured, as the distribution of each individual into the groups was performed in automated mode by the software, without researcher interference.

### Sample size

To determine the sample, the G*POWER 3.1.9.7 software was used, based on the study by Sadjapong et al. [31], which investigated the effect of multicomponent physical training on frailty syndrome. The frailty variable was used as the primary outcome for the sample size calculation, considering the difference between the means of two independent groups (baseline—24th week) and the repeated-measures ANOVA test, using Cohen’s f of 0.25 as the effect size, significance level of 5% (α = 0.05), and statistical power of 0.80, thus resulting in a minimum sample size of 34 participants. Although some studies have reported dropout rates between 20 and 25% as observed by Coelho-Júnior et al. [32], we conservatively chose a total number of 60 older adults due to the specific conditions of the sample, study duration, and greater reliability in data analysis.

### Experimental design

This study was conducted as a randomized, controlled, parallel clinical trial with two groups and assessments at three time points: pre-intervention (baseline), an intermediate assessment at 12 weeks, and post-intervention (immediately post-intervention at 24 weeks). After the screening process and application of eligibility criteria, each older adult was randomly allocated to one of the two groups: the experimental group, in which participants were submitted to the BWIT protocol, or the control group (CG), which represented a minimal intervention group, being instructed immediately after the baseline assessment to maintain physical activities in their daily routine during the study period. Blinding of participants, assessors, or the researcher responsible for the intervention was not possible due to the nature of the protocol.

### Assessments

The same standardized set of assessments was applied at the three time points of the study (pre-intervention, intermediate, and post-intervention), encompassing the evaluation of frailty status, physical-functional capacity, cardiohemodynamic responses, and body composition, with the intervention period beginning after the baseline assessment. The procedures were conducted by two previously trained researchers (principal investigator and research assistant).

### Sociodemographic data and body composition

Sociodemographic information was obtained through a structured questionnaire, including age, marital status, education, family income, occupational activity, and place of residence. Body weight was measured using a digital scale (Welmy®, model W200A, Brazil) and height using a stadiometer (Sanny®, model ES2030, Brazil). Body composition was assessed by electrical bioimpedance (InBody 120®), allowing estimation of fat-free mass, body fat in kg, and their respective percentages in the upper limbs, lower limbs, and trunk.

### Frailty

Frailty status was classified according to the phenotype proposed by Fried et al. [3], composed of five criteria: reduced handgrip strength (adjusted for sex and body mass index), reduced gait speed (time to walk 4.6 m adjusted for sex and height), unintentional weight loss (more than 4.5 kg or more than 5% of body weight in the last year), self-reported fatigue (identified by two questions from the Center for Epidemiological Studies Depression Scale [CES-D], considering as positive the report of exhaustion in at least one of the items with a frequency greater than three days in the last week), and low level of physical activity (assessed using the short version of the International Physical Activity Questionnaire [IPAQ], based on the estimation of weekly energy expenditure [kcal/week], applying sex-adjusted cutoff points according to the proposal of the phenotype Fried et al. [3]). Based on the number of criteria met, participants were classified as robust (0 criteria), pre-frail (1–2 criteria), or frail (≥ 3 criteria).

### Isometric knee extension and elbow flexion strength

Maximal isometric knee extension and elbow flexion strength were assessed using a portable dynamometer (Hand-Held Dynamometer – HHD; Medeor Medtech®, Brazil). For knee extension, the dominant limb was tested with the participant seated, knees at 90°, and the dynamometer positioned anterior to the ankle, approximately 5 cm above the lateral malleolus, stabilized with a belt to minimize evaluator interference. For elbow flexion, the dominant limb was evaluated in the seated position, with the elbow at 90°, confirmed by goniometer. In both tests, three maximal isometric contractions of 5 s were performed, with 3-min intervals between attempts, and the mean of the measurements was considered for analysis. Values were recorded in kilograms (kg).

### Five-times sit-to-stand test (5xSTS)

Functional performance (power) of the lower limbs was assessed by the five-times sit-to-stand test (5xSTS) as described by Guralnik et al. [33]. Participants were instructed to stand up and sit down five consecutive times, as quickly as possible, on a chair without armrests (45 cm height), keeping their arms crossed over the chest. Time was recorded from the initial elevation of the hips until completion of the fifth movement. Three attempts were performed, and the mean of the times was considered for analysis.

### Timed up and go (TUG)

Functional mobility was assessed by the Timed Up and Go (TUG) test [34]. Participants were instructed to stand up from a chair (45 cm height), walk 3 m, turn to return, and sit down again. Time was recorded from the initial command until the moment the participant rested their back against the chair backrest again. Three attempts were performed, and the mean of the times was considered for analysis.

### Bodyweight interval training (BWIT)

The experimental group (BWIT) performed a BWIT program consisting of 3 sessions per week, on alternating days, for 24 weeks. Each session was composed of 5 min of warm-up, 20 min of bodyweight interval exercise, and 5 min of stretching for cool-down. The protocol followed the proposal of Machado et al. [35], consisting of 20 cycles of 30 s of stimulus followed by 30 s of passive recovery (20 min in total). Intensity was monitored using the OMNI-Resistance Exercise Scale (OMNI-RES) [36], being predominantly maintained between 6 and 8 points, corresponding to moderate to vigorous efforts.

In the first month, the first week was dedicated to adaptation and familiarization, with intensity between 4 and 5 points on the OMNI-RES. From the second week onward, intensity was adjusted to 6 points, being maintained until the end of the second month. In the third and fourth months, it was increased to 7 points, and in the final two months, intensity was increased to 8 points, characterizing a linear progression of load throughout the intervention.

Intensity was autoregulated through adjustments in movement execution speed during stimulus periods, such that participants were instructed to accelerate or control movement execution in each exercise as the target perceived exertion value planned for each phase was reached. Thus, the number of repetitions performed in each cycle could be autoregulated according to the execution speed required to achieve the desired intensity for each participant, respecting the individual limit.

Informative intensity data were recorded during the second and fifth recovery intervals of each session. Sessions were structured in blocks [37], with four exercises selected per session and five cycles of stimulus and recovery per exercise. The selected movements, all adapted, included: seal jack, mountain climber, squat, squat thrust, squat jump, jump cross jack, and rowing sit-up. Training sessions were supervised and conducted by the principal investigator, a physical education professional with experience in exercise prescription for older adults. Over the 24 weeks, the principal investigator’s guidance evolved from detailed technical instruction during the familiarization week, based on demonstration of each movement and verbal instruction, to supervision focused mainly on observing execution and monitoring intensity and safety through verbal guidance, with technical teaching no longer being necessary. The BWIT sessions were conducted at the same location where participants were recruited, a primary health care unit named UBS Barcelona. Sessions were delivered in a group format within the unit’s premises, in a space with minimal structural resources and no equipment specific to physical exercise, requiring only the participants’ own body weight.

### Statistical analysis

Statistical analysis was conducted using R software (version 4.5.0) [38]. Initially, descriptive statistics were performed, expressed as mean ± standard deviation (continuous variables) and absolute and relative frequencies (categorical variables).

To evaluate the effects of training over time and between groups (CG vs. BWIT), linear mixed models with random intercept per participant were used, considering time (baseline, 12 weeks, and 24 weeks) as an intra-individual variable and group as an inter-individual variable. These models allow robust handling of repeated measures, unbalanced data, and missing values. The linear mixed models incorporated all observations with available data throughout follow-up. Analyses were performed with parameter estimation by restricted maximum likelihood (REML) and degrees of freedom adjusted by the Satterthwaite method.

The statistical significance of the main effects (group, time) and interaction (group × time) was tested using type III ANOVA. For continuous variables, effect sizes (partial η^2^) and their 95% confidence intervals were also calculated, classified according to conventional criteria (small < 0.06; moderate 0.06–0.14; large > 0.14).

For categorical variables related to frailty status (Fried classification), transition matrices (baseline → 12 weeks; 12 → 24 weeks) were constructed to identify individual changes between the categories “Frail,” “Pre-frail,” and “Robust.” Proportions of improvement between groups were compared using Fisher’s exact test or the chi-square test, when applicable.

Significance was considered for p values < 0.05. In addition to p values, results were interpreted taking into account effect magnitude (partial η^2^ for continuous variables and proportions of change for categorical variables).

## Results

A total of 97 older adults were assessed for eligibility. Of these, 37 were excluded, 21 due to unavailability to participate and 16 for not meeting the inclusion criteria. Sixty participants (n = 60) were randomized, with allocation of 30 to the control group (CG) and 30 to the BWIT group.

**Fig. 1:**
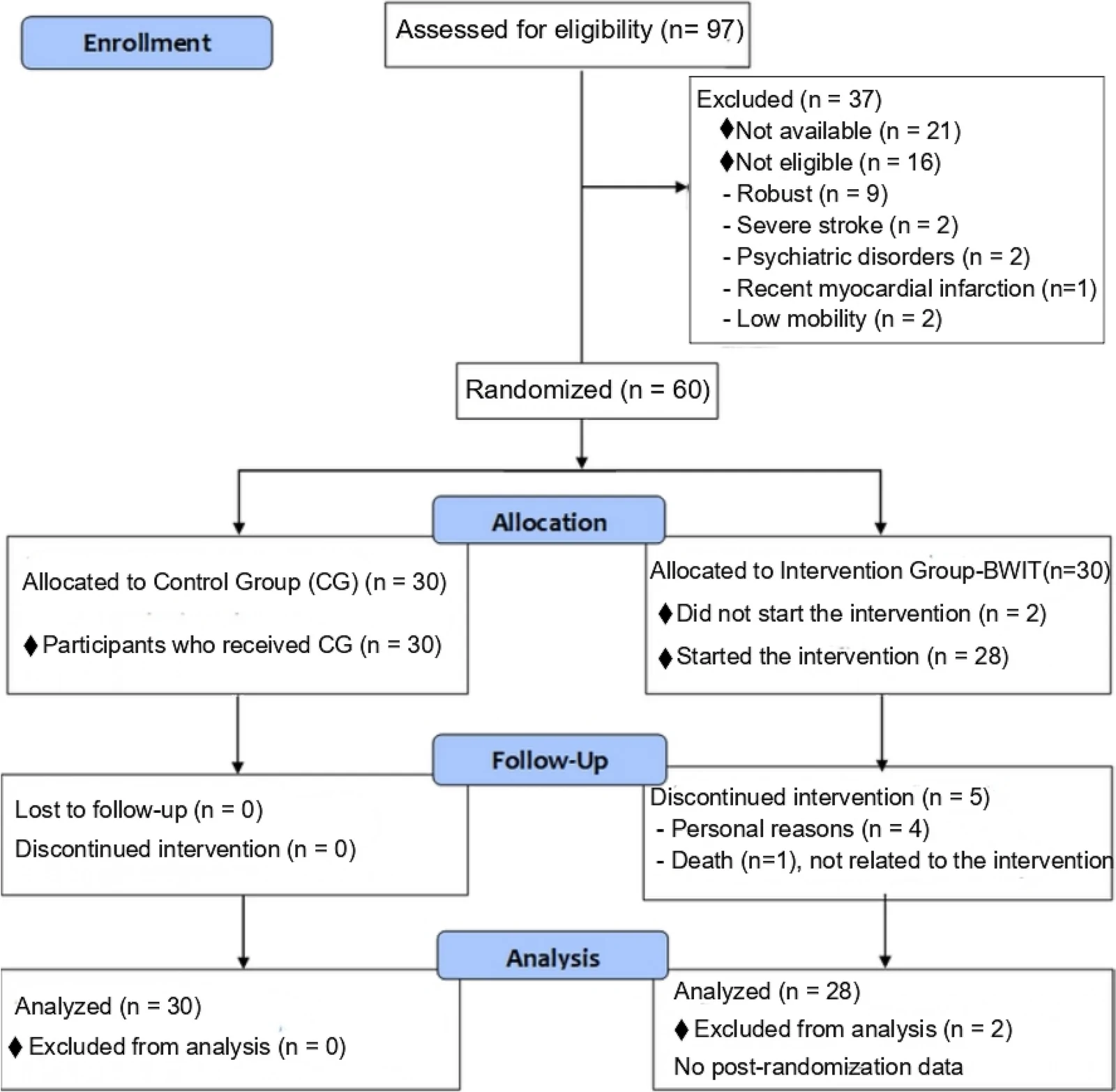
Consolidated Standards of Reporting Trials (CONSORT) flow diagram

In the CG, there were no losses to follow-up. In the BWIT group, two participants (n = 2) did not start the study after randomization and were therefore not included in the analysis. Among those who initiated the intervention, four (n = 4) discontinued for personal reasons and one (n = 1) participant died, not related to the intervention. These 5 participants were retained in the analysis. Thus, 28 participants in the BWIT group and 30 in the CG contributed data to the analysis. The participant flow throughout the study is presented in Fig. 1.

Baseline characteristics of the sample are presented in Table 1. Participants in this study had a mean age of 74.0 ± 6.0 years and were predominantly female (72.4%). No significant differences were observed between groups in demographic variables and in most body composition variables. However, the BWIT group presented significantly higher values of total body mass and appendicular skeletal muscle mass compared with the control group. The distribution of the frailty phenotype classification was similar between groups, although the frailty score differed at baseline, with a higher proportion of elevated scores in the BWIT group.

**Table 1:** General characteristics of the sample

Variables	Total^a^	Groups		p value^b^
		Control (n = 30)^a^	BWIT (n = 28)^a^	
Sex n(%)				
F	42 (100%)	23 (55%)	19 (45%)	0.45
M	16 (100%)	7 (44%)	9 (56%)	
Age (years)	74 (6)	74 (6)	74 (7)	0.73
Total body mass (kg)	66 (11)	63 (10)	69 (11)	0.034
Height (cm)	155 (152, 163)	155 (150, 162)	156 (153, 163)	0.59
Body mass index (kg/m^2^)	26.8 (4.1)	26.0 (3.8)	27.7 (4.3)	0.11
Body fat (%)	35 (29, 40)	35 (30, 40)	35 (28, 40)	0.67
Apendicular muscle mass (kg)	15.4 (13.9, 19.4)	14.8 (12.8, 18.0)	16.8 (14.5, 20.0)	0.041
Fried classification				
Frail	23 (100%)	11 (48%)	12 (52%)	0.63
Pre-frail	35 (100%)	19 (54%)	16 (46%)	
Fried score				
1	10 (100%)	8 (80%)	2 (20%)	0.039
2	25 (100%)	11 (44%)	14 (56%)	
3	13 (100%)	9 (69%)	4 (31%)	
4	7 (100%)	2 (29%)	5 (71%)	
5	3 (100%)	0 (0%)	3 (100%)	

^a^n (%); Mean (SD); Median (Q1, Q3)

^b^Pearson’s chi-squared test; Welch two-sample t-test; Wilcoxon rank-sum test; Fisher’s exact test

Two models were estimated to evaluate the evolution of frailty: an ordinal classification model and a continuous score model, as presented in Table 2.

**Table 2:** Results of the frailty models (ordinal classification and score)

Model	Variable/term	Estimate (OR ou β)	IC95%	p value
Ordinal classification	Frail → Pre-frail	2.17	0.49–9.62	0.306
	Pre-frail → Robust	1888	140–25.430	< 0.001
	BWIT group	15.44	2,17–110	0.006
	Time (12 weeks)	4.96	1.61–15.31	0.005
	Time (24 weeks)	15.24	4.15–55.93	< 0.001
Score	Intercept	2.17	1.80–2.53	–
	BWIT group	0.58	0.059–1.11	–
	Time (12 weeks)	− 0.008	− 0.27–0.26	–
	Time (24 weeks)	0.027	− 0.24–0.29	–
	BWIT group × Time (12 weeks)	− 1.55	− 1.94 to − 1.15	–
	BWIT group × Time (24 weeks)	− 2.11	− 2.51 to − 1.71	–
	SD (Intercept)	0.873	–	–
	SD (Observation)	0.526	–	–

Values expressed as odds ratios (OR) for the ordinal classification model and β coefficients for the score model, accompanied by 95% confidence intervals (95% CI) p values presented only for the ordinal model

In the ordinal classification model, the analysis showed that the Pre-frail → Robust transition was statistically significant (OR = 1888; 95% CI 140–25.430), whereas the Frail → Pre-frail transition did not reach statistical significance. Regarding the main effects, a significant association was observed both for the BWIT group (OR = 15.44; 95% CI 2.17–109.63) and for Time at 12 and 24 weeks.

Considering the score model, the intercept presented a mean value of 2.17 (95% CI 1.80–2.53). The main effects of BWIT group and Time were not statistically significant when considered in isolation. However, the BWIT group × Time interactions were strongly negative and significant, both at 12 weeks (β =  − 1.55; 95% CI − 1.94 to − 1.15) and at 24 weeks (β =  − 2.11; 95% CI − 2.51 to − 1.71).

**Fig. 2:**
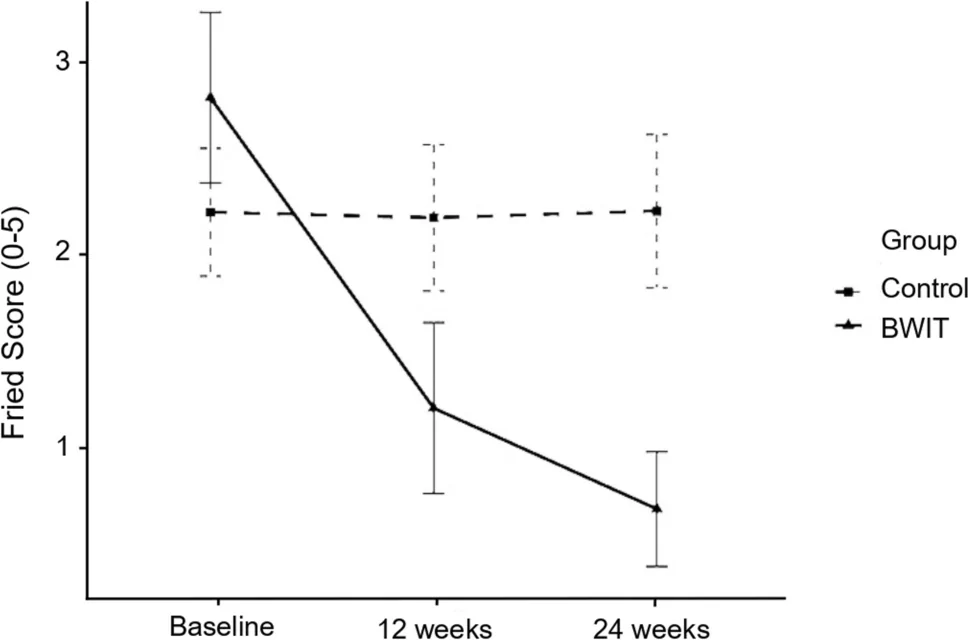
Evolution of the Frailty Score (Fried Score) in the CG and BWIT over 24 weeks

As shown in Fig. 2, the CG presented stability of scores across the three assessed time points. In contrast, in the BWIT group there was a reduction in scores between baseline, 12 weeks, and 24 weeks.

**Fig. 3:**
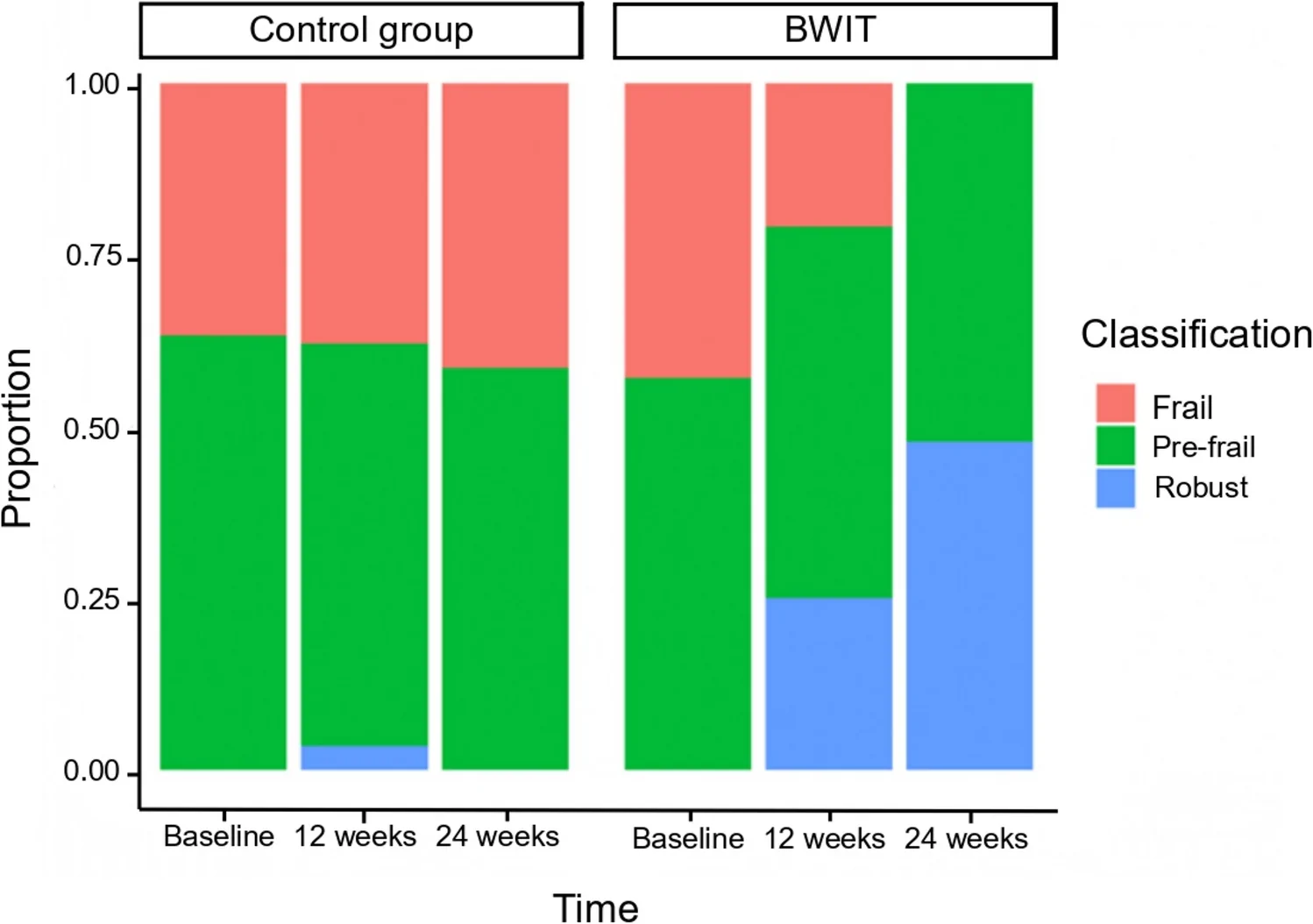
Distribution of frailty phenotypes between CG (n = 30) and BWIT (n = 28) over 24 weeks. Values presented as the proportion of participants in each category (Frail, Pre-frail, Robust)

Figure 3 presents the proportional distribution of frailty phenotypes throughout follow-up. In the CG, the distribution of categories remained relatively stable between baseline, 12 weeks, and 24 weeks. In the BWIT group, a progressive reduction in the proportion of participants classified as frail and an increase in the proportion of robust individuals were observed over 24 weeks. This visual pattern is consistent with the group × time interaction identified in the frailty models, suggesting transitions to less frail states in the group submitted to the intervention.

Transitions in frailty classification are presented in Online Resource 1. Between baseline and 12 weeks, a higher proportion of participants in the BWIT group presented transition to less frail categories compared with the control group (35.7% vs. 6.7%; p = 0.009). In the period between 12 and 24 weeks, additional favorable transitions were observed only in the BWIT group, with no events in the CG.

Values expressed as β coefficients and 95% confidence intervals (95% CI).

The ANCOVA results (Table 3), adjusted for baseline values, age, and sex, indicated a significant effect of the BWIT group for handgrip strength and knee extension strength. Age was negatively associated with leg strength (β =  − 0.14; 95% CI − 0.26 to − 0.02; p = 0.025). No significant effects were observed for elbow flexion strength or TUG, although male sex was associated with shorter execution time (β =  − 8.42; 95% CI − 16.22 to  − 0.61; p = 0.035). The BWIT group showed significant improvement in the 5xSTS and increased gait speed.

**Table 3:** Impact of the BWIT intervention on physical-functional capacity, cardiohemodynamic, and body composition variables

Domain	Variable	Group BWIT (β)	IC95%	p value
Physical-functional capacity	Handgrip strength (kg)	4.42	2.99–5.84	< 0.001
	Knee extension strength (kg)	6.69	5.15–8.23	< 0.001
	Elbow flexion strength (kg)	6.39	− 0.43–13.22	0.066
	5 × Sit-to-stand (s)	17.22	10.48–23.95	< 0.001
	Gait speed (m/s)	0.41	0.32–0.51	< 0.001
	TUG (s)	− 0.69	− 8.37–6.99	0.858
Cardiohemodynamic	SBP (mmHg)	− 7.49	− 11.08 to − 3.90	< 0.001
	DBP (mmHg)	− 5.99	− 8.51 to − 3.47	< 0.001
	MAP (mmHg)	− 6.60	− 8.90 to − 4.31	< 0.001
	Heart rate (bpm)	2.01	− 1.04 – 5.06	0.191
Body composition	Body weight (kg)	0.048	− 0.73 – 0.82	0.902
	Body mass index (kg/m^2^)	1.79	− 1.71 – 5.30	0.308
	Body fat (%)	− 0.72	− 1.88 – 0.44	0.215
	Appendicular muscle mass (kg)	0.23	− 0.058 – 0.512	0.115

In the cardiohemodynamic domain, significant reductions in systolic blood pressure, diastolic blood pressure, and mean arterial pressure were observed in the BWIT group, with no differences for heart rate. No significant differences were identified for body weight, BMI, body fat, or appendicular muscle mass.

### Longitudinal analysis

To evaluate the longitudinal trajectories of physical-functional, cardiohemodynamic, and body composition variables, linear mixed models with random intercept per participant were fitted, considering the effects of group, time, and group × time interaction.

In the physical-functional domain, there was a significant group × time interaction for isometric knee extension strength, 5xSTS, TUG, and gait speed.

For knee extension strength, although the BWIT group presented lower values at baseline (β =  − 3.10; p < 0.001), there was a significant group × time interaction favorable to BWIT at 24 weeks (β =  + 4.11; p < 0.001) (Online Resource 2).

Considering the 5xSTS, the BWIT group presented shorter execution time at 12 weeks (β =  − 3.57; p < 0.001), with an increased difference at 24 weeks (β =  − 1.37; p < 0.001), with no significant changes in the CG (Online Resource 3).

In the TUG test, there was an unfavorable baseline difference for BWIT (β =  + 2.31; p < 0.001), which was reversed over follow-up, with interaction at 24 weeks (β =  − 1.95; p < 0.001) (Online Resource 4).

For gait speed, the groups did not differ at baseline. At 12 weeks, the BWIT group presented higher speed compared with the CG (β = 0.250; p < 0.001), with an increased difference at 24 weeks (β = 0.16; p = 0.003), whereas the CG presented a significant reduction at 24 weeks (β =  − 0.098; p = 0.007) (Online Resource 5).

For appendicular muscle mass, no isolated effect of time was observed; however, the group × time interaction was significant only at 24 weeks (β = 0.22; p = 0.042), with a slight increase in BWIT.

In the cardiohemodynamic domain, there was a significant group × time interaction for MAP (p < 0.001; partial η^2^ = 0.30) and SBP (p < 0.001; partial η^2^ = 0.22), with significant reduction in the BWIT group over 24 weeks. For DBP, there was no significant interaction, although main effects of group and time were observed (F(1,56) = 662.51; p < 0.001; partial η^2^ = 0.92), indicating an increase over follow-up. Heart rate did not show a significant interaction.

No adverse events or exercise-related injuries were observed during the intervention period. Participants became familiar with the exercises during the first week of the intervention, and the low execution complexity of the movements may have facilitated this process.

## Discussion

This study was conducted in the context of primary health care and evaluated the effect of BWIT on frailty status, physical-functional capacity, cardiohemodynamic parameters, and body composition in older adults. The results suggest that BWIT was effective in promoting favorable modifications in frailty status in older adults, evidenced by different analytical approaches to the Fried phenotype. This intervention was associated with a higher probability of transition to less frail states, especially from pre-frail to robust, and progressive reductions in frailty score over 24 weeks, while the CG maintained its trajectory. In parallel, there were relevant improvements in physical-functional capacity and blood pressure parameters, with a slight increase only in appendicular muscle mass, but without significant changes in body composition.

The observed effects refer to the understanding of frailty syndrome as a dynamic condition potentially modifiable by structured interventions such as physical exercise. Convergent results were described by Tarazona-Santabalbina et al. [16], obtaining reversal of the frailty phenotype in community-dwelling older adults after 24 weeks of supervised multicomponent training, with 31.4% of participants becoming robust. In a protocol developed in a community context, as a supervised home-based program over 12 months, more favorable transitions between frailty categories were demonstrated, however with a moderate effect [39]. These studies corroborate that structured interventions can modulate the frailty trajectory in the context of primary health care as well as in the community; however, in this scenario, when evaluating frailty status the present study considered longitudinal evolution during follow-up, characterizing the dynamics of its transitions.

The comparison with Nagai et al. [40] offers additional perspective on the clinical relevance of the coherence between a higher probability of favorable transitions, reduction in score, and proportional redistribution of phenotypes related to frailty syndrome. The authors reported a significant reduction in frailty score after 24 weeks using resistance training combined with physical activity, however without modifications in frailty categories. In contrast, in this clinical trial BWIT promoted score reduction accompanied by clinically favorable transitions for the investigated condition and by the emergence of robust individuals, suggesting that the clinical translation of evolution may require sufficient magnitude and breadth to produce observable categorical changes.

For the ordinal model, BWIT was associated with a higher probability of transition to less frail states, especially from pre-frail to robust, and although the direction of the estimated effects indicates a clinically relevant effect, caution is required in interpreting its magnitude due to the wide confidence interval of the odds ratio. Regarding the pattern of greater probability of transition in this result, the category-dependent behavior in this study differed when compared with that described by Suikkanem et al. [39] and Coelho-Júnior et al. [32], both reporting that the main frailty-related transition in individuals was toward pre-frailty.

Although the duration of the BWIT intervention can be considered moderate, the magnitude of the score reductions related to the Fried et al. [3] frailty criteria observed in the present study is considered relatively more pronounced than in other studies [39] describing longer interventions. Furthermore, while the CG maintained a stable pattern, the integrated analysis reinforces interpretations related to the effect of BWIT, since the observed reductions were determined exclusively by the group × time interaction, and not by isolated effects of group or time, suggesting not only attenuation with respect to statistical improvement in score, but a structural modification in the clinical phenotype of the older adults participating in the intervention.

Other results reported in recent studies, depending on intervention characteristics, assessment instruments, and application context, demonstrated reversal of frailty in institutionalized older adults after multicomponent training [18], while an intervention conducted [41] in a primary health care setting was effective in reducing frailty prevalence, showing benefit in only 3 months of intervention with a combined approach of physical exercise and nutrition. In turn, in this conducted trial, a protocol exclusively based on bodyweight exercises and follow-up over 24 weeks suggested promoting progressive improvement in frailty, with potential applicability in primary health care contexts.

In addition to the effects on frailty status, BWIT promoted improvements in outcomes that represent central dimensions of functionality and physical independence in older adults, and constitute direct or indirect components associated with the Fried et al. [3] phenotype.

Favorable outcomes in higher levels of muscle strength and power have been associated with frailty reversal in older adults after resistance training programs [32], and additional evidence suggests that resistance training can consistently improve strength, sit-to-stand performance, and Short Physical Performance Battery (SPPB) scores in pre-frail and frail older adults [19, 42]. It is suggested that BWIT was effective in activating similar mechanisms, such as neuromuscular adaptations, even without the use of external loads or traditional physical training methods. The clinical relevance of these results can be highlighted by the fact that the 5xSTS test and gait speed reflect central dimensions of independence in older adults [43]. Complementarily, usual gait speed is also a robust prognostic marker, with progressive increases associated with greater survival in older adults, and increments of only 0.1 m/s already presenting clinical relevance, giving meaning to the favorable response in the BWIT group compared with the CG [44].

In the cardiohemodynamic domain, BWIT also promoted relevant responses in cardiohemodynamic parameters, outcomes related to cardiovascular risk and morbidity and mortality in older adults, and recognizedly associated with frailty. The MAP and SBP parameters, characterized by sustained reductions throughout follow-up and by significant group × time interactions, contrasted with the stability observed in the CG, demonstrating a pattern coherent with evidence according to Sardeli et al. [45], who reported that blood pressure reductions induced by physical training in older adults are also determined by dependence on baseline blood pressure levels, being more pronounced when individuals present elevated values initially. Regarding body composition, with a slight increase in appendicular muscle mass, no significant changes were observed in the other parameters, without characterizing relevant hypertrophic gains, suggesting that the improvements observed in physical-functional capacity and frailty status possibly resulted from mechanisms not dependent on quantitative increases in muscle mass, consistent with clinical studies with frail older adult participants, which demonstrate preservation of lean mass even in the presence of clinical and functional benefits [16, 41].

We highlight the need to consider the limitations presented by this study. The reduced sample size, as well as recruitment in a single context and the absence of post-intervention follow-up to evaluate the maintenance of the observed effects. We acknowledge the absence of blinding among the assessing researchers and the researcher responsible for the intervention, although this is a challenge due to the type of intervention. On the other hand, despite the study application context, the non-inclusion of direct measures of physiological mechanisms and of dietary or nutritional control represents limiting factors. Furthermore, even with randomization, the BWIT group presented higher baseline total body mass and appendicular muscle mass than the CG, which imposes limitations for more specific inferences, since these characteristics may influence the capacity to respond to training. The generalizability of the findings should be interpreted with caution for populations with lower treatment adherence and a higher risk of frailty, such as older adults with mild cognitive impairment, depressive disorders, or social barriers that limit participation in face-to-face exercise programs, including limited transportation access and financial support.

Among the strengths of this study, we present the study design associated with longitudinal assessment and its complementary forms of analysis. In addition, we highlight the intervention format, based on a bodyweight exercise protocol, representing low logistical complexity and financial costs and, therefore, accessible in diverse contexts of care for older adults.

From a practical standpoint, several aspects of the BWIT intervention may influence its implementation, as it required no specialized infrastructure or equipment, relying solely on body weight, and was delivered in a group format by a single professional at a potentially low cost, in the physical space of the primary health care unit. Its delivery, however, depended on a qualified professional to teach the exercises and supervise intensity and safety, although the availability of such professionals may represent the main constraint to implementation in settings with limited human resources. A progressive transition toward partially self-managed or home-based practice, supported by periodic professional contact, may represent a pathway for wider dissemination and be evaluated in future studies.

In conclusion, the present findings suggest that bodyweight interval training was effective in promoting clinically relevant effects on modifying frailty status in older adults in primary health care, accompanied by physical-functional gains and reductions in blood pressure parameters over 24 weeks. In contrast, no relevant changes in body composition were observed, suggesting that functional and regulatory adaptations resulting from the intervention promoted the benefits found. These results indicate that a bodyweight exercise protocol could potentially constitute a strategic intervention in settings with structural and resource constraints, being accessible for the management of frailty in primary health care. In this sense, further studies are suggested to investigate the dose–response of bodyweight interval training and its effectiveness in different stages of frailty, as well as to evaluate physiological mechanisms associated with bodyweight training in frail older adults. Future longitudinal studies could also examine whether the benefits of bodyweight interval training extend to major clinical outcomes, such as hospitalization, institutionalization, and mortality.

## Supplementary Information

Below is the link to the electronic supplementary material.

Supplementary file1 (PDF 99 KB)

Supplementary file2 (PDF 212 KB)

Supplementary file3 (PDF 124 KB)

Supplementary file4 (PDF 182 KB)

Supplementary file5 (PDF 121 KB)

## Data Availability

The datasets analyzed during the present study are available from the corresponding author upon request.
