# Supplementary material for: Effect of bodyweight interval training on frailty status in older adults: a randomized controlled trial
Source: Eur Geriatr Med. 2026 Aug 12;17(5):2919–30. doi: 10.1007/s41999-026-01579-8 (PMC13755087; doi:10.1007/s41999-026-01579-8)
Supplement: Supplementary file 4 [file 41999_2026_1579_MOESM4_ESM.pdf]

Effect of bodyweight interval training on frailty status in older adults: randomized controlled trial

European Geriatric Medicine

Lucas Marques Vieira [1]* (ORCID: 0009-0001-7010-1224)
Danilo Sales Bocalini [2] (ORCID: 0000-0003-3993-8277)
Clarcson Plácido Conceição dos Santos [3] (ORCID: 0000-0001-7598-3775)
Leandro Brasil Rego [1] (ORCID: 0000-0001-9559-9770)
Rodrigo Villar [4] (ORCID: 0000-0001-8568-3346)
Aylton José Figueira Júnior [5] (ORCID: 0000-0001-8069-2366)
Rodrigo Chaves da Silva [1] (ORCID: 0009-0006-2910-469X)
Ana Paula de Souza Lima [1] (ORCID: 0009-0003-8115-8749)
Ruy Barbosa Martins Calheiros Netto [1] (ORCID: 0009-0007-8670-0946)
Felício Savioli Neto [6] (ORCID: 0009-0009-9773-7570)
Taina Costa Nonato [1] (ORCID: 0009-0008-0048-8671)
João Paulo Pinto [1] (ORCID: 0009-0007-8870-5150)
Francisco Luciano Pontes Júnior [1] (ORCID: 0000-0002-8358-0260

[1] School of Arts, Sciences and Humanities, University of São Paulo - Exercise Physiology and Aging Laboratory (EACH-USP), São Paulo, SP, Brazil
[2] Federal University of Espírito Santo, Vitória, Espírito Santo, Brazil
[3] Bahiana School of Medicine and Public Health, Salvador, Bahia, Brazil
[4] University of Manitoba,Winnipeg, Canadá
[5] São Judas Tadeu University, São Paulo, São Paulo, Brazil
[6] Dante Pazzanese Institute of Cardiology, São Paulo, São Paulo, Brazil

Corresponding author:
Lucas Marques Vieira
Email: lucasmarquesprof@gmail.com

Online Resource 4

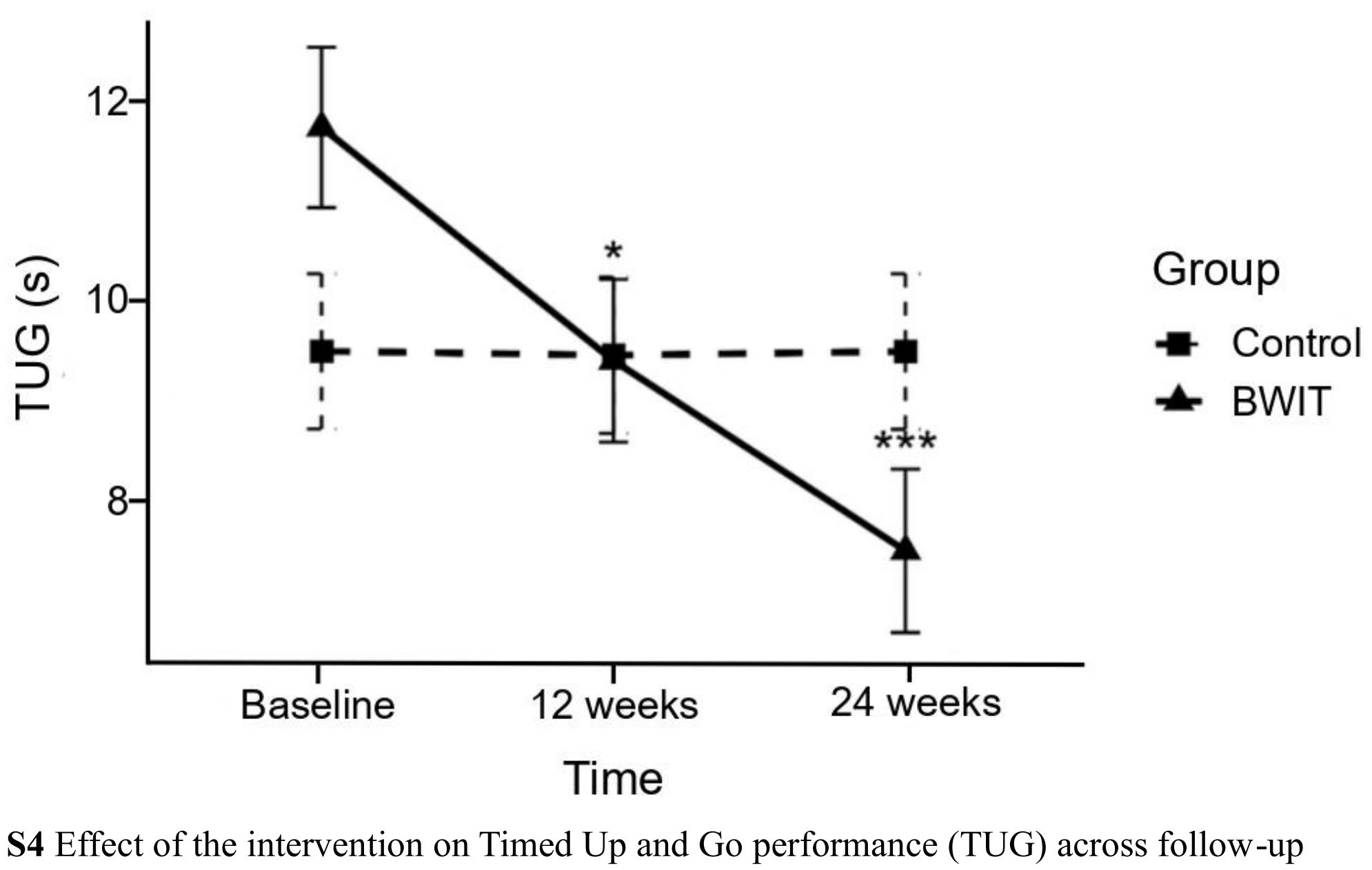

Fig. S4 Effect of the intervention on Timed Up and Go performance (TUG) across follow-up

Note: Intergroup differences at each time point are indicated by asterisks (* $p < 0.05$; *** $p < 0.001$). Abbreviations: BIT, bodyweight interval training group; CG, control group; TUG, Timed Up and Go
